# Design of Economic Evaluations of Mindfulness-Based Interventions: Ten Methodological Questions of Which to Be Mindful

**DOI:** 10.1007/s12671-014-0282-6

**Published:** 2014-02-01

**Authors:** Rhiannon Tudor Edwards, Lucy Bryning, Rebecca Crane

**Affiliations:** 1 Centre for Health Economics and Medicines Evaluation (CHEME), IMSCaR, Bangor University, Bangor, LL57 2PZ UK; 2 Centre for Mindfulness Research and Practice (CMRP), School of Psychology, Bangor University, Bangor, LL57 1UT UK

**Keywords:** Health economics, Economic evaluation, Cost-effectiveness, Trial design, Mindfulness-based interventions, Meditation, Costs

## Abstract

Mindfulness-based interventions (MBIs) are being increasingly applied in a variety of settings. A growing body of evidence to support the effectiveness of these interventions exists and there are a few published cost-effectiveness studies. With limited resources available within public sectors (health care, social care, and education), it is necessary to build in concurrent economic evaluations alongside trials in order to inform service commissioning and policy. If future research studies are well-designed, they have strong potential to investigate the economic impact of MBIs. The particular challenge to the health economist is how best to capture the ways that MBIs help people adjust to or build resilience to difficult life circumstances, and to disseminate effectively to enable policy makers to judge the value of the contribution that MBIs can make within the context of the limited resourcing of public services. In anticipation of more research worldwide evaluating MBIs in various settings, this article suggests ten health economics methodological design questions that researchers may want to consider prior to conducting MBI research. These questions draw on both published standards of good methodological practice in economic evaluation of medical interventions, and on the authors’ knowledge and experience of mindfulness-based practice. We argue that it is helpful to view MBIs as both complex interventions and as public health prevention initiatives. Our suggestions for well-designed economic evaluations of MBIs in health and other settings, mirror current thinking on the challenges and opportunities of public health economics.

## Introduction

Mindfulness-based interventions (MBIs) are being increasingly applied in a variety of settings. Typically an MBI is a group-based, 8-week programme consisting of a series of mindfulness meditation practices led by a trained ‘mindfulness-based teacher’. Internationally, the most renowned interventions are mindfulness-based stress reduction (MBSR; Kabat-Zinn 1990) and mindfulness-based cognitive therapy (MBCT; Segal et al. 2002). Published evidence on the effectiveness of these and other MBIs has increased over the last 15 years (Cullen 2011; Williams and Kabat-Zinn 2011). MBCT has strong evidence of effectiveness in reducing relapse in recurrent depression (Piet and Hougard 2011) and is recommended as a priority treatment for people with recurrent depression by the National Institute for Health and Care Excellence in the UK (NICE 2004; 2009). MBCT also has growing evidence of effectiveness in other areas including anxiety (Evans et al. 2008), insomnia (Heidenreich et al. 2006) and bipolar disorder (Williams et al. 2008a). MBSR has demonstrated effectiveness in a range of physical and mental health conditions and with a range of population groups (see Bohlmeijer et al. 2010; Chiesa and Serretti 2009; and Grossman et al. 2004 for reviews). Increasingly, programmes are being developed from MBSR and MBCT to promote resilience in general healthy populations in schools (e.g. Kuyken et al. 2013), for parents (e.g. Bailie et al. 2012) and in workplaces (e.g. Chaskalson 2011).

Early proponents of MBIs argued it to be a ‘cost-effective’ alternative to one-to-one psychological therapy or in the case of management of depression, pharmacological intervention (Segal et al. 2002; Teasdale et al. 2000). This was a logical hypothesis, due to MBIs being routinely offered in the UK and USA as group-based interventions to between 8 and 30 participants. However, the cost-effectiveness of an intervention can only be evaluated in relation to an alternative intervention of a lower cost and/or greater effectiveness (Brazier et al. 2007; Drummond et al. 2005). For example, an approach should not automatically be considered cost-effective because it is delivered in large groups at low average cost per participant. Any effect may be small or short-lived. Robust health economics research is necessary to establish cost-effectiveness of any interventions.

Worldwide, mindfulness research is at a point of rapid expansion and application (Williams and Kabat-Zinn 2011), and there has been a call for more health economics research (Edwards and Bryning 2013; Kabat-Zinn 2013). This article suggests ten methodological design questions that those researchers responsible for the design of the health economics component of MBI trials could usefully consider. Health economists have been charged with being ‘dispassionate and unbiased’ (Culyer 2012), but also we argue, need to be fully informed in appropriate research design and knowledgeable about the intervention being evaluated, and specific context in which it is being evaluated. We argue that this is very much the case in MBI research. Well-designed economic evaluation, alongside future trials, offer the potential for generalisable, unbiased, and detailed information about the potential costs and benefits of MBIs and are more likely to usefully inform commissioning of health care, social care, education and well-being policies by governments and other regulatory bodies.

We propose that the economic evaluation of MBIs (even in a clinical setting) benefits from perspectives drawn from research on public health interventions because the methodological challenges in both contexts are similar. It is challenging to capture the range of effects of public health interventions because they are often about trying to modify individuals’ behaviours and as such are sensitive to the setting in which interventions take place. These settings are often synergistic with other socio-economic considerations and causality is complex and difficult to define (Kelly et al. 2005). Furthermore, MBIs are increasingly being offered to healthy populations as well-being, resilience-building strategies.

These are not new methodological considerations, but they are presented here with specific consideration of their application to the evaluation of MBIs. The questions are based on a well-known checklist of characteristics of good economic evaluations (Drummond et al. 2005). They are further informed by a checklist for health economics papers for publication in the *British Medical Journal* (BMJ 1996).

The ten methodological design questions are as follows:

1. *What is the specific research question being asked about the MBI under evaluation*?
  We suggest researchers formalise their health economics research question in terms of “How cost-effective is the MBI, for people with ‘x’, in the setting ‘y’, as compared with ‘z’ as usual practice”. An intervention cannot be cost-effective if it is not first clinically effective. We acknowledge that in stating this we are operating within the ‘medical model’ paradigm of Evidence-Based Medicine first proposed by Archie Cochrane (1972). This medical model relates survival and health-related quality of life more to medical interventions received, than to the socio-economic circumstances in which someone lives or psychosocial influences on health-related quality of life. His contemporary and a founding father of British health economics, Alan Williams, argued that it was unethical to devote scarce health (or social care) resources towards interventions that deliver very low benefits per currency unit (e.g. £, $ or €) spent, when those same resources have an opportunity cost (Williams 1974). This opportunity cost is the value of resources in their next best use that could have been devoted to the delivery of another potentially more effective intervention, for the same group of individuals or for other individuals, in another setting. In consideration of this research question, we introduce concepts of efficiency (getting the most from available resources), equity (fairness in access to treatment), moral considerations (ethics) and the central concept in health economics of opportunity cost (the forfeit of benefits in their next best use). It is this final concept that distinguishes health economists from accountants or financial planners. We consider costs in their fullest sense of what we forgo in choosing one use of scarce resources over another. In most Western societies, health care and social care systems are funded through tax or insurance, and the central or collective pot or budget is limited. Even in the USA which spends more on health care per capita and more on health care as percentage of its GDP (17.9 % in 2011) than any other nation (OECD 2011; WHO 2013), resources are limited to what individuals, employers and the government are prepared to devote to health care over and above other uses such as education, housing, the environment and defence.
  MBIs can be considered complex interventions, as defined by the Medical Research Council in the UK (MRC 2008). They have many inter-related components, which may individually or synergistically account for benefits observed in research (e.g. curriculum, group format, characteristics of therapist/teacher, fidelity of programme delivery). Mindfulness itself as a construct is difficult to define (Grossman 2008; Grossman and Van Dam 2011), and it is challenging for researchers wishing to distil out specific mechanisms of effectiveness and cost-effectiveness. The MRC in the UK has published a guide to the evaluation of complex interventions which is a useful resource to those designing feasibility and/or full trials of MBIs (MRC 2008). This guide outlines the need to clearly ‘define’ a complex intervention, and to this end the MBI needs to be specifically described and documented (e.g. with a manual) to allow for comparisons with a control condition and to assess factors such as intervention fidelity and adherence (Chiesa et al. 2011). The research question should define the intervention, the population and the setting for the purpose of evaluating cost-effectiveness before introducing the alternative with which it will be compared
2. *With what alternative intervention or situation is the MBI to be compared*?
  Randomised controlled trials (RCTs) are considered to be the gold standard within which to assess effectiveness and cost-effectiveness (Kendall 2003). There are many different designs of trials and not all studies of MBIs will be in trial settings. However, in order for evidence of the effectiveness of an MBI to be considered on an equal plane by the medical and clinical professions, RCTs are necessary, with opportunities to build a concurrent economic evaluation within such a trial (Ramsey et al. 2001).
  Where possible, MBI research should be within a RCT setting. However in many settings, MBIs are being delivered ahead of the evidence and without routine rigorous evaluation (Bishop 2002) and thus we recognise that in these cases this research should adopt a pragmatic approach that embraces the real-world nature of the delivery and uptake of MBIs (Edwards et al. 2008). Pragmatic trials are conducted in the community, in full knowledge that the setting and the outside world is likely to impact on trial outcomes, but that with an appropriate sample size and randomisation will be able to demonstrate a difference in effect between the intervention being studied and the control condition (Hennekens and Buring 1987).
  The control condition needs to be a clinically relevant alternative or, as is conventional, reflect ‘usual practice’, which sometimes can mean no active treatment. In a trial of the management of relapse of depression, Kuyken et al. (2008) compared MBCT plus maintenance anti-depressants (mADM) with mADM alone. This trial was designed to reflect wide spread ‘usual care’ and acknowledged MBCT as a component of care rather than a straightforward alternative to pharmacological management.
  In the evaluation of psychosocial interventions, especially group-based interventions, which contain an element of ‘socialisation’, there may be some beneficial effect of just meeting (Karasu 1986), and factors such as this be considered in documenting, describing and standardising the control condition in a trial of a MBI. A number of studies have used active control groups within the trial design of MBI evaluation (Grossman et al. 2007; Zautra et al. 2008). In a protocol of a trial, which aims to dismantle the various components of MBI to explain the mechanism of an effect, the active control condition, Cognitive Psycho Education was described and was designed to include all components of MBCT except mindfulness meditation practice (Williams et al. 2010). This trial aims to establish whether MBCT is effective in preventing relapse into depression for people who become suicidal when depressed. It is as important to collect not only costs of the intervention arm of a trial but also costs of the control arm, to allow for calculation of an incremental cost-effectiveness ratio (ICER).
3. *How is effectiveness to be measured*, *and what is an important change on such measures*?
  As in the evaluation of many clinical interventions, the primary outcome measure needs to be selected and there is a need to consider what constitutes a ‘clinically important or significant’ change on the chosen outcome measures. It may be that mindfulness training cannot change the prognosis of a disease such as cancer, but instead provides psychological support for patients dealing with diagnosis, treatment and the overall experience of cancer (Shennan et al. 2011). The outcome measure to determine clinical effectiveness therefore needs to reflect the area of expected change.
  To date, the most compelling evidence for MBI has been with the management of recurrent depression. There is evidence for reduction in rates of depression relapse (Piet and Hougaard 2011). Kuyken et al. (2010a) outlines the mechanism by which MBCT achieves this preventative effect through changing the participants’ relationship to the experience of depression. Rather than fighting to prevent a depression relapse, participants develop a compassionate and interested relationship with the negative thoughts and emotions that can precipitate a depression episode. This radical shift in attitude and approach creates a range of other well-being-enhancing effects beyond protection from depression. Researchers are increasingly working to capture the diversity of effects both through process evaluation integrated within RCTs (e.g. Williams et al. 2010) and through qualitative evaluations which can capture the subtleties of an approach (e.g. Allen et al. 2009).
  For comparison across studies, it is useful to choose commonly used questionnaires such as, in the case of the management of depression, the Hospital Anxiety and Depression Scale (Zigmond and Snaith 1983). This supports service commissioners reviewing evidence to interpret the relative benefit of achieving an improvement on the chosen outcome measure. While using the same measures as those used in other studies aids comparability, it also has the disadvantage of narrowing the range of outcomes that are being evaluated and valued. Researchers therefore need to also include measures which capture the particular shift in relationship to experience that is the core focus of mindfulness-based training (Baer et al. 2011). These are increasingly being included in large trials (e.g. Kuyken et al. 2010b; Williams et al. 2010) using measures of mindfulness (Five Factor Mindfulness Questionnaire; Baer et al. 2006), self-compassion (Self-Compassion Scale; Neff 2003), rumination (Ruminative Responses Subscale of the Response Styles Questionnaire; Treynor et al. 2003), dysfunctional attitudes (Dysfunctional Attitudes Scale; Oliver and Baumgart 1985) and acceptance (Acceptance and Action Questionnaire; Hayes et al. 2004).
  The early trials of MBIs were very small (Grossman et al. 2004) and underpowered (Baer 2003). The power of a trial is the extent to which we are able to detect a ‘real’ difference between the intervention and control condition, and not just a difference that might come about as a result of random difference in the population (Altman 1990). Powering is also important to trials of MBI because trials will often by their nature be what are called ‘cluster randomised trials’ with each group of participants constituting a cluster as randomisation takes place at group level rather than individual level, perhaps with a waiting list control (Williams et al. 2008b). The dynamics of delivering an intervention in a group context need to be acknowledged when planning powering. The researcher must consider whether one MBI group is the same as another, with a different teacher, a different room and held at a different time of day. This will determine the number of study participants necessary to detect a true difference in outcomes of interest between arms of the trial. Powering is important to economic evaluation in that we are concerned with joint distributions of costs and effects for each participant in a trial. It is well accepted in health economics that cost data is skewed to the right (Briggs and Gray 1998). This is due to the fact that in any sample of patients or individuals the majority of observations are likely to be low in cost; however, characteristically there will be a few individuals who are very high consumers of health and social care. Ideally, a robust economic evaluation needs to be based on an even larger sample size than that needed to show clinical power (Briggs and Gray 1998). This rarely ever happens in clinical trials of any intervention. Sample size is in practise determined by clinical outcomes and limitations to the research design due to ethical considerations and research funding opportunities. It is the task of the health economist within a multi-disciplinary trial team, to simultaneously bring attention to the accurate measurement of costs *and* to the appropriate measurement of outcomes or benefit.
4. *Which economic analysis method is most appropriate and from who*’*s perspective is the MBI evaluation being conducted*?
  Traditionally, health economists have distinguished between several methods of analysis. Drummond et al. (2005) outlines these as cost–benefit (which measures costs and benefits in monetary terms), cost-effectiveness (which measures outcomes in some appropriate natural unit), cost-utility (which measures outcomes in some universal measure of health gain, e.g. the quality adjusted life year QALY) and cost-consequence analysis (that compares costs with a full range of disaggregated outcomes).
  In order to determine the method of economic evaluation that is most appropriate and the range of benefits and costs to be collected, it is necessary to identify from whose perspective costs and benefits are being evaluated. As MBIs are increasingly being delivered in non-clinical settings such as schools and workplaces, there is an increasing imperative to move away from the ‘medical model’ paradigm (which in the UK would traditionally involve an NHS perspective in economic analysis, with a limited range of costs and benefits collected relevant to the NHS) and move towards a broader public sector multi-agency perspective (Edwards et al. 2008). This wider perspective allows for greater recognition of the need to collect a diverse range of costs and benefits across different sectors.
  Cost-effectiveness analysis usually requires choice of a single primary end point of a clinical trial as its measure of effect (e.g. relapse rate in the management of depression). Cost-utility analysis requires a preference-based measure of health-related quality of life and it is conventional to use a generic, non-disease-specific measure to allow for cost per Quality Adjusted Life Year (QALY) estimates. The QALY is an index of the additional years of life gained from a medical or other intervention, adjusted in some way to reflect health-related quality of life (Robinson 1993).
  Cost–benefit analysis, closest to a welfarist approach, demands that we measure all costs and benefits in monetary terms from a societal perspective. Historically, this has infrequently been used in health economics as it is difficult to express health benefits in monetary terms. Though the few trials with concurrent economic evaluations have adopted a cost-utility or cost-effectiveness approach in a clinical setting (Kuyken et al. 2008; van Ravesteijn et al. 2013), cost–benefit analysis may be the answer for capturing the benefits of MBIs in non-health care settings such as schools and workplaces. In the case of researchers interested in measuring the costs and benefits of mindfulness training in education or in the workplace, capturing the financial impact of improving educational standards or reducing absenteeism may be an effective way of measuring benefits. In addition to cost–benefit analysis, cost-consequence analysis has been recommended by those tackling the methodological challenges of public health interventions, as a way of capturing a full range of benefits, to the individual, family, setting, school or workplace and wider society (Kelly et al. 2005; Weatherly et al. 2009). Weatherly et al. (2009) describes the need to consider spill over effects, referred to by economists as ‘externalities’. For example, a parent who is coping better with depression through MBCT may be a more effective parent and in turn benefits may be observed in their children’s behaviour. The education sector may therefore receive some of the benefit of a public health intervention delivered by the health sector. It is in considering this that well-being becomes the concern of all government sectors and supports a shift from a medical model paradigm of health to an integrated ‘whole systems’ perspective. It may be that cost-consequence analysis is also the most appropriate design for an economic evaluation of an MBI. However, it is important to note that cost-consequence analysis does not allow for a simple comparison with other interventions as is possible with cost-per-QALY estimates and may not be appropriate when a clear decision rule is required (e.g. in health care). In principle, a trial of MBI in a setting or patient group where generic measures have been shown to be sensitive, e.g. management of depression (Sapin et al. 2004; Sobocki et al. 2007), and where a threshold is relevant for a funding decision as in the case of NICE in the UK (NICE 2008, 2013), a cost-utility approach may be more appropriate. A cost-utility analysis or cost-effectiveness analysis embedded in a wider cost-consequence analysis may meet commissioners need to inform a decision rule, whilst acknowledging the wider range of outcomes from a MBI.
5. *How are costs of a MBI to be measured*? *And what is the range of costs to be considered*?
  Micro-costing is the bottom-up construction of the costs of a programme, treatment or intervention. Micro-costing can involve careful specification of training costs, staffing costs, venue overheads, materials and staff travel, where appropriate. Micro-costing techniques have been used effectively in similar group-based interventions such as group parenting programmes (Charles et al. 2013) and are appropriate in an MBI context. Micro-costing is currently being used to determine the full costs of delivering MBIs in different settings, for example in supporting patients receiving treatment for cancer (Edwards and Bryning 2013).
  Costing the delivery of an MBI may involve costing training of the teacher (and these costs can be annuitised over an appropriate period, e.g. 3–5 years, when the teacher might be expected to continue teaching); running costs, including ongoing supervision of teachers and attendance on continuing professional development training; room hire; overheads; materials such as books and CDs for home practice; and administrative support. Many teachers may subsidise the cost of delivering an MBI through personally incurring the costs of ongoing training and supervision, which may be viewed as personal development and deepening of their personal mindfulness practice (Edwards and Bryning 2013). On the basis that teachers’ time does have an alternative use, these costs should be calculated and included in the economic evaluation. This principle applies to such things as room hire, which may appear to be ‘free’ (for example a bookable room in a hospital, school or workplace), and it is necessary that their market costs are included either in the base case analysis or in sensitivity analysis to illustrate how costs of running an MBI vary under different assumptions, for example, about rates paid to teachers or the number of teachers in each group.
  According to the principles of economic evaluation, all resources with alternative uses within the chosen perspective need to be measured and valued. Where MBIs are being delivered within a health and/or social care setting as well as identifying the costs of delivery, it is necessary to capture the impact of service use by individuals following an intervention. It is important to assess whether MBIs actually lead to any increase in appropriate service use (through information and contact gained or recognition and acceptance of problems), substitution or reduction of service use, or a reduction in reliance on services (such as the family doctor).
  The DIRUM database (www.dirum.org) provides a repository of resource or service use instruments appropriate for different clinical and non-clinical settings (Ridyard and Hughes 2012). Capturing resource use and associated costs enables health economists to draw conclusions about the impact of MBIs on demands on traditional health and social care services. These broader societal findings could be important to those wanting to make a case for the funding of MBIs in future and potential integration into primary care, school or workplace.
6. *How should benefits of a MBI be measured for an economic evaluation*?
  With respect to MBIs, the challenge facing health economists relates, firstly, to capturing the benefits of helping people accept or adjust to difficult life circumstances and promote resilience and, secondly, to relating these benefits to limited resources in health and other public services. A cost-utility approach requires use of a generic preference-based utility measure which considers both impact on life expectancy and health-related quality of life.
  MBIs used to help people with severe depression and suicidal thoughts may contribute to improving life expectancy across a trial population (Williams et al. 2006). MBIs may improve health-related quality of life across existing life expectancy in many different settings and for a range of health conditions or life circumstances.
  In the UK, the National Institute of Health and Care Excellence supports the use of the EQ-5D (EuroQol Group 1990) in health economic evaluations of interventions and health technologies (NICE 2013). EQ-5D is a validated generic, health-related, preference-based measure comprising five domains: mobility, self-care, usual activities, pain and discomfort, anxiety and depression. Each domain has three levels (no problems, some/moderate problems and extreme problems). The EQ-5D scoring system defines 243 (3^5^) possible health states with two additional states (dead and unconscious), where death has a value of 0 and best imaginable health has a value of 1. The questions are complemented by a thermometer style, visual analogue scale, with 0 representing worst imaginable health and 100 representing best imaginable health, on which respondents are asked to indicate their current health state. EQ-5D has the benefit of being short, clear and quick to complete. The more recent introduction of a five-level version of the EQ-5D which includes the addition of slight and severe problems to each domain (EQ-5D-5L; Herdman et al. 2011) may deliver improved performance while still retaining the benefit of brevity, consisting of just five questions (Scalone et al. 2012). As the number of economic evaluations of MBIs increases, it will be interesting to see whether researchers choose to include the EQ-5D-3L or EQ-5D-5L, and whether this generic instrument proves sufficiently sensitive to pick up the change in approach and attitude to life that people undertaking mindfulness-based training may experience. The EQ-5D has been successfully used in trials of major depression (Sapin et al. 2004; Sobocki et al. 2007). For example, the question “I can undertake my usual activities” may at first appear to be directed purely at the physical functioning of the individual and does not discern how the individual is relating to their functioning; however, it is important to note that our psychological functioning may also influence our ability to undertake these usual activities. While mindfulness-based training may not directly influence functional capacity to undertake usual activities, it is likely to affect the level of ease with which the individual lives within their current capacities (Kuyken et al. 2010a). An individual who is at ease is more likely to be able to seek and accept appropriate levels of support and less likely to suffer from psychological distress in relation to their functional capacity (Kuyken et al. 2010a).
  We suggest that those designing an economic evaluation of an MBI do include EQ-5D or another generic alternative such as the SF-6D (Brazier et al. 2002) or HUI (Horsman et al. 2003) to allow comparability with clinical trials of other relevant psychological interventions or in the case of depression, pharmacological interventions (Brazier et al. 2007). Another emerging option is that of the ICECAP measures (Grewal et al. 2006; Al-Janabi et al. 2012). The ICECAP-O, developed for older adults, adopts a capabilities approach and is intended to be a more encompassing quality of life measure than the QALY (Coast 2004). There are five domains: attachment, security, role, enjoyment and independence and four levels of capability (ranging from a lot to none; Grewal et al. 2006). The development of the ICECAP-A for adults again adopts a capabilities approach however is suitable for use with younger populations (Al-Janabi et al. 2012). The ICECAP-A aims to measure factors relevant to an adult population rather than older adults and identifies five domains: stability, attachment, autonomy, achievement, and enjoyment and four levels of capability (Al-Janabi et al. 2012).
  Randomised trials are needed to establish whether the conventional research instruments such as EQ-5D and SF-6D are sufficiently sensitive to reflect such potential benefits. In a trial of MBCT for medically unexplained symptoms conducted in the Netherlands, the resultant QALY gains were very small leading to an ICER of Euro 57,000 per QALY (van Ravesteijn et al. 2013). This is significantly above the threshold of what society feels is an appropriate investment to gain a QALY, as operationalised by decision making bodies such as NICE in the UK (NICE 2013). Further methodological research is needed to compare QALY gains using EQ-5D or SF-6D with disease-specific measures which are relevant to the context in which the MBI is being delivered (Brazier et al. 2010). At this stage of health economics research in the field of evaluation of mindfulness interventions, we encourage the inclusion of generic-, clinical- and intervention-specific outcome measures where possible to allow for methodological enquiry.
7. *Are the study results sensitive to changes to our assumptions*?
  Sensitivity analysis is used in economic evaluation to allow the researcher to vary the base case analysis to explore how sensitive results are to changes in key parameters, e.g. in a health care setting: length of hospital stay, grade of staff or dose of a drug (Drummond et al. 2005). A key issue in economic evaluation of group-based psychological therapies is dose. If people do not attend all classes or a defined number of classes (as outlined in the trial protocol), they do not get a ‘sufficient dose’, and this may affect both the average cost of an intervention, and potentially outcomes. The challenge for the health economist is to define the ‘base case’ for analysis. In the case of MBIs, these are the assumptions underpinning the main analysis, for example, the number of individuals attending a class and the number of classes constituting a MBI programme. Health economists have developed a range of more sophisticated probabilistic sensitivity analysis techniques which aim to further address uncertainty, going beyond using mean values for key parameters such as costs, clinical outcomes and utility values, and instead use the full distribution of these parameters to estimate uncertainty (Brazier et al. 2007).
  Concerns about generalisability from a trial setting lead us to consider whether the setting and context of a trial determine its findings and extent to which those findings may be replicated more widely in practice. This is very much the case when considering societal or health equity considerations.
8. *Does the research consider equity as well as efficiency*?
  It is important that those studying the effectiveness and cost-effectiveness of MBIs in different settings give equity due consideration. Public health practitioners are concerned with equity or inequalities in health and a central goal has often been to improve the health of the worst off in society. Health economists are increasingly recognising the need to incorporate equity considerations into their analysis (Weatherly et al. 2009). This represents a departure from traditional economic analysis which often implicitly has focused on an underlying extra welfarist paradigm of health gain maximisation for society (Culyer 2012). This represents a practical operationalisation of a ‘trade-off’ between the goal of efficiency (i.e. maximising an outcome such as health gain) and goals of improving equity which may mean a sacrifice of total potential health gains to society. Worldwide, 20 % of people may experience mental health problems during their lifetime. Mental health disorders are estimated to account for a third of all years lived with a disability (McDaid et al. 2008). Mental health problems including suicide are linked with socio-economic status (Department of Health 2009). The UK Department of Health recommends that all policies relating to health, including mental health, should be assessed in terms of their potential impact on inequalities in health, and where possible, favouring the less well off (Department of Health 2009). It is important that those designing studies of the effectiveness and cost-effectiveness of MBIs consider that impacts on equity has implications for the generalisability of results and is key to the question of how to alleviate suffering associated with those facing multiple challenges of living in socio-economically challenging circumstances.
  As the number of trials of MBIs increase and their settings become more varied, this aspect of economic evaluation will come to the fore. This brings us to the potential longer term impact of mindfulness-based training and the role of modelling in economic analysis.
9. *Is economic modelling going to be useful in MBI research*?
  Economic modelling is used by health economists, particularly in the pharmacological industry, as an alternative to economic evaluation alongside a clinical trial using patient level data, particularly where there is a need to extrapolate beyond the length of follow-up (Briggs et al. 2006). Modelling allows consideration of uncertainty, particularly probabilistic decision analytic modelling. It involves drawing estimates of costs and estimates of effectiveness and the probabilities of patients/individuals moving from one health state to another, e.g. from episodes of wellness to relapse in depression, in a Markov model (Briggs and Sculpher 1998).
  However, in order to construct meaningful models, sufficient data is required. There are at present very few trials of MBIs that include an economic evaluation component and, even when those underway now are published, there will still be relatively few. There is a need to consider that cost and outcome findings from an MBI in one setting, e.g. cancer care, cannot necessarily be extrapolated to a completely different setting, e.g. workplace well-being. More trials and economic evaluations are needed in a range of settings.
10. *How should results be compared with the findings of others studies*, *and used to advise service commissioners and policy makers*?
  There is a growing body of evidence relating to psychological therapies/psychosocial interventions to support better mental health in schools, workplaces and in health care settings (Knapp et al. 2011). MBIs have a place amongst these psychological therapies both in terms of treatment, prevention and promotion of resilience.
  What is now needed is a body of robust trial-based evidence of effectiveness and cost-effectiveness to help service commissioners and health and social care policy makers, and in other contexts those responsible for education policy and for improving social capital, to consider the relative value for money of different psychological therapies, delivered in different settings, and reaching different groups in society. This is where building cost-per-QALY estimates as well as condition-specific outcome measures provides an opportunity for service commissioners and policy makers to compare and rank interventions in terms of their value for money (NICE 2008).

## Discussion

While there is a growing body of evidence supporting effectiveness of MBIs, more well-designed research evaluating cost-effectiveness is now needed. Specific considerations towards the design of a research trial or study are necessary in order to provide a robust body of evidence for service commissioners allocating scarce health care and social care resources. Many of the challenges facing the economic evaluation of public health interventions are relevant to mindfulness research. MBIs are complex interventions and it is important that the depth and breadth of effects are captured in economic analyses. Key methodological considerations are required to counter factors such as the need for a larger sample size as a result of a cluster randomised design. Researchers should choose the method of economic analysis most appropriate for the evaluating of MBIs based on the perspective that costs and benefits are being measured. Where a threshold is relevant for a funding decision, as in the case of NICE in the UK (NICE 2008), a cost-utility approach which allows for cost-per-QALY estimates and comparisons across interventions may be most appropriate. Cost–benefit analysis may be the most appropriate way for researchers interested in mindfulness training in school or workplace settings, to capture the full economic benefits of improving well-being in a broad sense. It may be that a cost–benefit approach is more appropriate than a traditional cost-effectiveness approach focussing on individuals’ assessment of their own health-related quality of life. Cost-consequence analysis is recommended by those interested in the methodological challenges of public health interventions as a solution to capturing a wide range of benefits, e.g. to an individual, family, school or work place, or wider society. It may be that cost-consequence analysis is most appropriate for capturing and illustrating the full range of wider benefits of MBIs where monetary values are difficult to assign to outcomes, as is necessary in cost–benefit analysis. A cost-utility analysis or cost-effectiveness analysis, embedded in a wider cost-consequence analysis, may meet commissioners need to inform a decision rule, whilst acknowledging the wider range of outcomes from a MBI. Public health practitioners focus on equity considerations and inequalities in health. It is important that those studying the effectiveness and cost-effectiveness of MBIs in different settings also give these due consideration, particularly with respect to making MBIs accessible to hard to reach groups in society. This is relevant to the generalisability of results and to the question of how to alleviate suffering associated with those facing multiple challenges of living in socio-economically challenging circumstances. While economic modelling may be useful in mindfulness research, especially to extrapolate into the longer term and to address uncertainty, more trials and data is needed in order to populate the parameter estimates required in economic models. The field of health economics has tended to operate within the medical model paradigm of quantitative analysis. However, more recently health economists have begun to acknowledge the potential contribution of qualitative research methods in explaining measurable relationships (Coast 1999). The health economist must be objective and dispassionate and want to find out whether an intervention is effective and cost-effective, regardless of whether the results of the trial or economic evaluation end up positive or negative. In our view, some appreciation for the purpose, origins, delivery and potential range of relevant costs and outcomes is essential to the design of a robust economic evaluation of MBIs. Existing published health economics literature of mindfulness interventions is beginning to inform the body of economic evidence in this area. We encourage future trials of MBIs to build in a concurrent economic evaluation in order to provide robust evidence to service commissioners.
